# Yawn Contagion and Empathy in *Homo sapiens*

**DOI:** 10.1371/journal.pone.0028472

**Published:** 2011-12-07

**Authors:** Ivan Norscia, Elisabetta Palagi

**Affiliations:** 1 Centro Interdipartimentale Museo di Storia Naturale e del Territorio, Università di Pisa, Calci, Pisa, Italy; 2 Unità di Primatologia Cognitiva, ISTC-CNR, Roma, Italy; University of New England, Australia

## Abstract

The ability to share others' emotions, or empathy, is crucial for complex social interactions. Clinical, psychological, and neurobiological clues suggest a link between yawn contagion and empathy in humans (*Homo sapiens*). However, no behavioral evidence has been provided so far. We tested the effect of different variables (e.g., country of origin, sex, yawn characteristics) on yawn contagion by running mixed models applied to observational data collected over 1 year on adult (>16 years old) human subjects. Only social bonding predicted the occurrence, frequency, and latency of yawn contagion. As with other measures of empathy, the rate of contagion was greatest in response to kin, then friends, then acquaintances, and lastly strangers. Related individuals (r≥0.25) showed the greatest contagion, in terms of both occurrence of yawning and frequency of yawns. Strangers and acquaintances showed a longer delay in the yawn response (latency) compared to friends and kin. This outcome suggests that the neuronal activation magnitude related to yawn contagion can differ as a function of subject familiarity. In conclusion, our results demonstrate that yawn contagion is primarily driven by the emotional closeness between individuals and not by other variables, such as gender and nationality.

## Introduction

Humans, the primates with the most complex social networks [1], rely on the ability to share others' emotions to engage in successful social interactions [2]. This phenomenon, known as empathy, relies on a perception-action mechanism [3]. The involuntary re-enactment of an observed behavior may arise in the observer by recruiting neural mechanisms that, during the perception of an action or of a facial expression, activate shared representations [3]–[5]. Contagious yawning, evoked by the yawn produced by a conspecific and widely demonstrated in human and non-human primates [6]–[15], also involves a similar action-perception mechanism [3].

Different clinical, psychological, and neurobiological clues suggest a link between yawn contagion and empathy. Contagious yawning starts occurring at 4–5 years of age [16], when children develop the ability to identify other's emotions properly [2], [17], [18]. Also, contagion is impaired in subjects suffering from empathy disorders, such as autism [19]–[21], and is positively related with self-reported scores of empathy (based on self-face recognition and *faux-pas* theory of mind tasks) [22]. Additionally, different neuroimaging studies converge in supporting the empathic basis of contagious yawning [23]–[25]. Posterior cingulate and precuneus activations when viewing someone yawning suggest that contagion involves empathy networks [23]. The negative covariance between amygdalar activation and subjective yawn susceptibility supports the relationship of yawn contagion and the face-processing-related emotional analyses during social interactions [24]. The activation of the ventromedial prefrontal cortex (involved in the empathic processes [26], [27]), associated with the urge to yawn by contagion, further suggests a relationship between contagion and empathy [25]. Finally, although evidence is controversial [23], mirror neurons in the right posterior inferior frontal gyrus might be recruited for contagion [28]. Mirror neurons are important for action understanding, a prerequisite for empathy [29].

In an evolutionary perspective, empathy is probably rooted in the emotional contagion characterizing the strongest of the family bonds, the mother-infant one [2], [3], [30]. The perception-action model predicts that in social species, individuals that require a response are those that a subject relies upon to attain personal goals, usually friends and kin. Thus, nervous systems that respond automatically with empathy to situations where they must respond maximize inclusive fitness [3]. This is why empathy is more pronounced the closer the relationship between individuals [3], [31]. Indeed, empathy and degree of closeness are correlated such that the magnitude of the response follows a pattern of kin > close friends > acquaintances > strangers [32].

So far, we present the only naturalistic study of yawn contagion in humans that provides evidence of the linkage between yawn contagion and empathy by demonstrating that yawn contagion i) is influenced by the social-emotional bond between individuals more than by any other variables considered (e.g. position, gender, social context, nationality differences) in terms of occurrence, frequency, and response latency; and ii) follows the same trend of empathy, thus increasing from strangers to kin.

## Results

Observations were performed over 1 year (2010/2011) and involved 109 adults (>16 y.o.), 56 females and 53 males from Europe, North America, Asia, and Africa, in their natural settings. During each observation period (spanning 6 min – 2 hours), yawning episodes were collected via the all occurrences sampling method [33]. When a subject yawned (the trigger), we recorded 1) time; 2) the encoded identity of the yawner (hereafter, the “trigger”) and of each potential responder (hereafter, the “observer”), that is every person in auditory and/or visual contact with the trigger (within 3 m); 3) triggering yawn characteristics (from the observer's perspective): i) sensory modality (auditory only; visual only; both visual and auditory); ii) number of yawns within 3 min; and iii) position of the observer (no visual contact; frontal to the trigger; diagonal); 4) presence/absence of contagion within 3-min following the last triggering yawn; 5) time latency in the yawn response to the trigger; 6) trigger's and observer's sex; 7) social bond (0 = strangers, 1 = acquaintances, 2 = friends, 3 = kin); 8) social context (work; feeding time; spare time; confined space (means of transportation); and 9) trigger's and observer's country of origin, then coded at the dyadic level (same country; different country). We recorded a total 613 bouts, then restricted to 480 because analyses only involved bouts in which the response yawns could be clearly assigned to a specific trigger (single-yawn trigger or a single trigger performing multiple yawns within the 3-min time slot).

**Figure 1:**
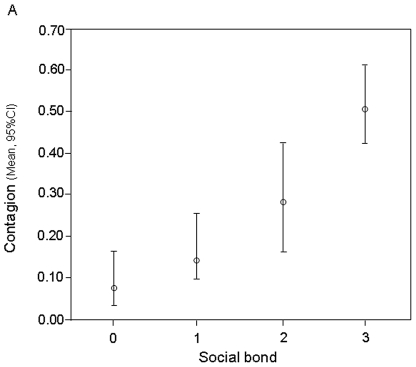
Contagion occurrence as a function of social bond. Model-estimated value of contagion (marginal means, Y axis), for each value of the main effect (social bond, X axis). Bars show the 95% upper confidence interval (95% CI) for the marginal means. GLMM (AICc = 2272.933; *n* = 480). Social bond categories: 0 =  strangers; 1 = acquaintances; 2 =  friends; 3 = kin with r≥0.25 and life partners).

Via a Generalized Linear Mixed Model (GLMM) we verified which variables affected the occurrence of yawn contagion (presence/absence; *n* = 480). Trigger's and observer's sex (and combination), number of triggering yawns, social bond, context, yawn sensory modality, position (and combination between sensory modality and position) and dyad country matching were entered as fixed variables. Among such variables, the only factor remaining in the best model (AICc = 2245.493) was the dyadic social bond (Table 1), which had a strongly significant effect on yawn contagion (F = 17.957, df1 = 3, df2 = 476, *P*<0.001). The presence of contagion was much higher when the social bond was closer (Table 1; Figure 1).

**Table 1:** Best GLMM explaining the occurrence of yawn contagion (AICc = 2272.933).

	Co	SE	t	p	95% CI
Intercept	0.076	0.201	0.378	0.705	−0.319/0.470
**FF**					
SB (0)	−2.490	0.433	−5.745	<0.001	−3.342/−1.639
SB (1)	−1.743	0.313	−5.565	<0.001	−2.359/−1.128
SB (2)	−0.938	0.303	−3.095	0.002	−1.534/−0.343
SB (3)	0^a^				
**RF**	Variance	SE			
Trigger identity	0.092	0.139			
Observer identity	0.023	0.092			

a redundant coefficient. Co: coefficient: SE: standard error; 95% CI: Confidence Interval; SB: Social Bond; FF: Fixed Factors; RF: Random Factors.

**Figure 2:**
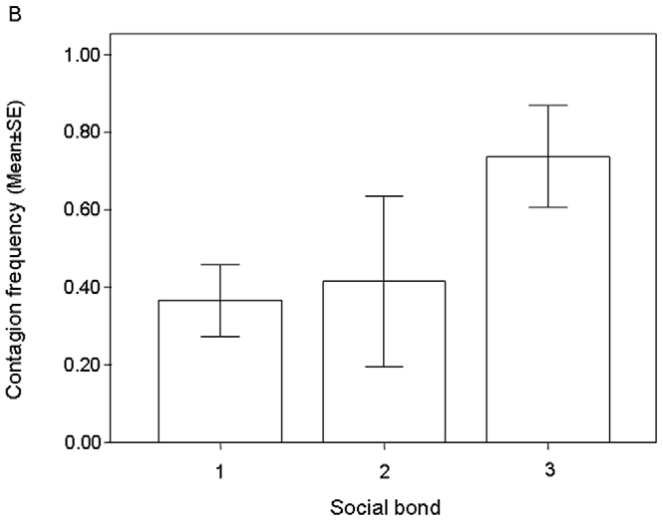
Bar graph of contagion frequency (mean±SE) for dyads of non-stranger subjects (social bond: 1-3). Mean±SE, 95% CI for each category: Bond = 1: 0.386±0.058, 0.267/0.505; Bond = 2: 0.519±0.080, 0.356/0.682; Bond = 3: 0.850±0.064, 0.720/0.979. LMM (AICc = 0.007, *n* = 48). Social bond categories: 1 = acquaintances; 2 =  friends; 3 = kin with r≥0.25 and life partners).

Via a LMM we verified which variables could explain the variation in the frequency of yawn contagion. Trigger's and observer's sex (and their combination), and social bond were entered as fixed factors. This analysis involved only those dyads (*n* = 48) where contagion had occurred (occasion opportunities≥3). Dyads of strangers were excluded. Only social bond remained in the best model (AICc = 0.007) positively affecting the frequency of contagion (F = 30.360, numerator df = 2, denominator df = 25,044, *P*<0.001), which increased alongside the tightness of the social bond (Figure 2; observer's identity variance ±SE: 0.041±0.019).

Via a Generalized Linear Mixed Model (GLMM) we verified which variables influenced the time latency in the yawn response by an observer to the trigger. This analysis involved only those bouts (*n* = 149) where contagion was present. Trigger's and observer's sex (and their combination), social bond, context, yawn sensory modality, position (and combination between sensory modality and position) and dyad country matching were entered as fixed variables. Again, the only factor remaining in the best model (AICc = 1116.604) was the dyadic social bond (Table 2), which had a significant, and negative effect on the response latency (F = 2.297, df1 = 6, df2 = 141, *P* = 0.038), with latency increasing as social bond closeness decreased. In particular, dyads with bond 0 (strangers) and 1 (acquaintances) showed a significantly higher latency in the yawn response to the trigger (Table 2).

**Table 2:** Best GLMM explaining the latency in the yawn response to a trigger (AICc = 1116.604).

	Co	SE	t	p	95% CI
Intercept	1.025	0.390	2.626	0.010	0.253/1.796
**FF**					
SB (0)	−2.815	1.179	−2.387	0.018	−5.147/−0.484
SB (1)	−1.531	0.733	−2.087	0.039	−2.980/−0.081
SB (2)	−0.009	0.636	−0.013	0.989	−1.265/1.248
SB (3)	0^a^				
**RF**	Variance	SE			
Trigger identity	0.590	0.790			

a redundant coefficient. Observer's identity variance is 0 so it is not indicated. Co: coefficient: SE: standard error; 95% CI: Confidence Interval; SB: Social Bond; FF: Fixed Factors; RF: Random Factors.

## Discussion

**Figure 3:**
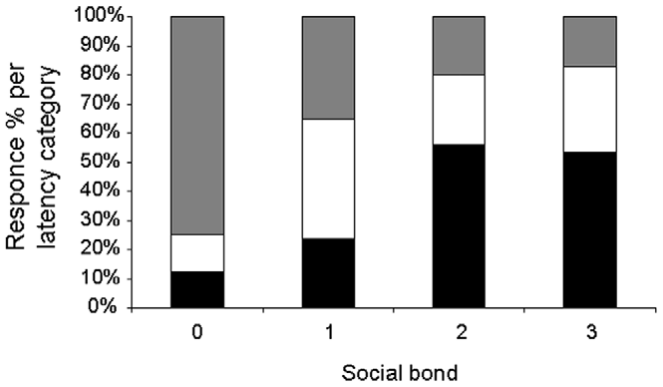
Yawn response latency as a function of the social bond. Stacked histograms displaying the repartition of yawn response % per each latency category (Y axis) within each social bond category (X axis). Response latency categories: 0 = 0<t_r_≤1 min (black); 1 = 1<t_r_≤2 min (white); 2 = 2<t_r_≤3 min (grey). Social bond categories: 0 =  strangers; 1 = acquaintances; 2 =  friends; 3 = kin with r≥0.25 and life partners). Social bond has a significant effect on response latency in the best model (GLMM, AICc = 1116.694, *n* = 149).

Here, we demonstrated that the social bond, associated with empathy [3], affects the yawn contagion in humans in terms of occurrence (Figure 1), frequency (Figure 2), and response latency (Figure 3).

Social bond overrode social context and nationality differences in explaining the occurrence of contagion and the variation in the response latency. Indeed, yawning is performed by all members of the human species, immediately recognizable, and occurring in all contexts [15], [34]. Thus, it is not surprising that yawn contagion is not seriously affected by context or country of origin.

Gender differences in the empathic abilities have been widely reported, with women showing higher empathy levels than men (e.g., [35]–[37]). Such differences should reflect in dissimilar yawn contagion levels of the two sexes, not revealed by our results (sex was also excluded from the best model). However, another analytical approach is needed for this purpose. In fact, possible gender divergences can only be revealed by considering yawn contagion of dyads belonging to the same social bond category (strangers to kin) and/or by examining the variation trend in the yawn contagion as a function of the social bond within each sex category.

In agreement with previous works, sensory modality did not affect contagion. In 1942, Moore [38] first reported that some blind subjects yawned in response to an audio recording of yawns. Arnott et al. [28] found that the sound of a yawn, like the sight of someone yawning, was effective at eliciting an urge to yawn and activated part of the mirror neuron area (the right posterior inferior frontal gyrus; pIFG). Additionally, simply reading about yawning is sufficient to trigger yawns, when no sensory cue is involved [13].

The lack of an effect of the position of the observer with respect to the trigger (frontal, diagonal, or lateral) on yawn contagion matches with Provine's [14], [39] observation that yawn-detection process is not axially specific; yawns in orientations of 90°, 180°, and 270° were as potent or nearly as potent as normal, upright, 0° yawns. Moreover, in patients with unilateral destruction of the visual cortex, Tamietto et al. [40] found evidence that emotional contagion occurs also when the triggering stimulus cannot be consciously perceived because of cortical blindness.

Contagion insensitivity to sensory modalities and to visual perspective (relative position) and consciousness clearly indicates that the stimulus quality does not play a primary role in triggering the yawning response in the observer. Some authors have questioned that attention differences (with observers paying closer attention to familiar subjects rather than to unfamiliar ones) could account for differences in the yawning response [41]. However, heightened arousal (degree of physiological responsivity relative to a baseline) is normally detected in response to novelty whereas diminished arousal is observed in response to perceived familiarity, as a part of the habituation process, an evolutionary adaptation to avoid an unbearable overloading of the attentional system [42].

The importance of social bond in shaping yawn contagion demonstrates that empathy plays a leading role in the modulation of this phenomenon. Not only is contagion greater between familiar individuals, but it also follows an empathic gradient [3], increasing from strangers to kin-related individuals. Such a gradient holds for both the contagion occurrence (presence/absence; Figure 1) and the entity of the response to a given trigger (frequency; Figure 2). This is the behavioral confirmation of what clinical, psychological, and neurobiological works have been suggesting over the past decade [22], [34].

Our findings go further in explaining the linkage between empathy and yawn contagion. In fact, the delay in the yawn response is longer when the trigger is less familiar to the observer (Figure 3). Perceiving other persons yawning activate a complex network of brain regions related to motor imitation, social behavior, and empathy, which also involves both sensorimotor cortices and limbic and para-limbic structures [2], [34]. Thus, the neural regions linked to the emotional sphere of positive affect may be over-stimulated in subjects viewing the yawn of someone they care about. Such over-stimulation may ultimately lead to a potentiated yawning response. A recent study [43] which investigated the response of smiles in mother-infant dyads supports this “over-stimulation hypothesis”. The results showed increased activation around the orbitofrontal cortex (OFC) in mothers viewing their own infant's smile compared to an unfamiliar infant's smile. The neuronal processing of positive affect can encompass different types of social interactions, from the mother-infant one to kinship, friendship, and romantic relationships [43]. In this case, specific neuronal regions involved in positive affect regulation are activated by both viewing familiar and unfamiliar subjects (infants) but the activation magnitude differs, being greater when social attachment is higher and familiarity are involved [43]. A neuro-ethological approach, similar to that used for smiles, should be applied to detect whether the neural pathways of yawn contagion differ as a function of the emotional closeness shared by the first yawner and the responder.

In an evolutionary perspective, the ability to replicate others' yawns, demonstrated in monkeys [11] is probably “older” than empathy, only found in human apes and only implied in bonobos and chimpanzees [44]. Via yawn replication, social animals can synchronize the behavioral and physiological state of a group [15]. However, replication becomes contagion when there is some evidence that an emotional transfer, requiring complex cognitive abilities, is involved. Hence, it is not surprising that the demonstration of a direct link between yawn contagion and emotional closeness in humans follows the trend observed in other primates [9], [11]. This is in line with the bottom-up perspective proposed by de Waal and Ferrari [45], who claim that a cognitive continuity bridges non-human to human primates. Indeed, emotional contagion represents an instance of truly affective reactions that may be mediated by neural pathways of old evolutionary origin providing a cornerstone for emotion communication and affect sharing [43]. Yawn contagion has been proven greater between group members (compared to extra group ones) in *Pan troglodytes* [9], and subjects sharing good relationships (measured via grooming) in *Theropithecus gelada* [11]. When considered together, these results suggest that the relationship between yawn contagion and empathy may have developed earlier than the last common ancestor between monkeys, human, and non-human apes.

## Methods

### Ethics statement

This study was purely observational (with no manipulation whatsoever) and information was entered in an anonymous form (individual data were entered under an alphanumerical code uniquely assigned to each subject). Moreover, the study subjects were observed in their natural social setting. Thus, the ethics committee of the University of Pisa waived the need for a permit.

### Data collection

Data collection was blind, with the observed subjects not aware of being under investigation. The study subjects were observed during their everyday activity, in their natural social setting (at work, in restaurants, waiting rooms, during social meals, etc.). In public spaces, the authors sat down close to the study subjects and observed. Subjects included people known to the authors, such as friends, family members, coworkers, and students, who could either know each other or not. The study also included individuals that the authors did not know but whose personal information (e.g., country of origin and social bond with other study subjects) was stated by the observed subjects during conversations. Data (in the form of alphanumerical codes) were typed and stored in mobile phones (e.g., during dinners), entered in the laptop (when possible, e.g., on the train), or noted down on paper (e.g., in public spaces where this practice could easily go unnoticed).

Yawns were observed at morning (7:00 am – 01:00 pm), afternoon/evening (01:00 pm – 07:00 pm), and night (07:00 pm –01:00 am). Yawn response can be evoked up to 5 min after observing another subject yawning [13], with a peak within 3 min [15]. As a cautious measure we recorded yawn responses on a 3-min time slot, thus reducing the probability of coding as a contagion response what, in fact, could be a spontaneous yawn. The two following minutes were excluded to reduce the probability of coding as spontaneous yawn (trigger) what, in fact, could be a contagion response.

To avoid bias linked to variable contagion frequency distribution along the day [10], observation time was adjusted to balance contagion bouts across the three daily periods (contagion bouts: χ^2^ = 1.91, df = 2, *P* = 0.385). Trigger-observer dyads were observed from a minimum of 30 min to a maximum of 2 h.

The occurrence of contagion was coded as: 1 = presence, 0 = absence; trigger's and observer's sex were labeled as: 1 = male, 2 = female; trigger's and observer's country of origin were coded at the dyadic level: 1 = same country; 2 = different country. Sensory modality was coded as: 0 =  auditory cue only, 1 =  visual cue only, 2 =  visual and auditory cues. Observer's position was defined as: 0 =  no visual contact; 1 = frontal to the trigger; 2 = diagonal, requiring a 45° head rotation to reach the frontal vision of the trigger; 3 = lateral, requiring a 90° head rotation to reach the frontal vision of the trigger.

The social bond was collected on four levels: 0 = strangers, who had never met before; 1 =  acquaintances, who exclusively shared an indirect relationship based on a third external reference, that is work duty (colleagues) or friends in common (friends of friends); 2 =  friends, non related individuals sharing a direct relationship, frequenting each other because they are willing to; 3 =  regular partners and kin (r≥0.25). The relationship between people was known to the authors. Ambiguous cases where excluded from the dataset (e.g. kin with r<0.25, colleagues frequenting each other outside work).

The social context was categorized as follows: 1 = work; 2 = feeding time; 3 = spare time; 4 =  confined space (means of transportation).

The response latency (t_r_) was measured as the time delay between the last trigger's yawn and the response by the observer, scored on three levels: 0 = 0<t_r_≤1 min; 1 = 1<t_r_≤2 min; 2 = 2<t_r_≤3 min.

Two observers were concurrently present during data recording and alternatively noted down the information as a short alphanumerical string on paper (when possible) or by typing it on a mobile phone. Before starting systematic data collection, reliability between observers was tested during a 10-day trial period. At the end of the period, Cohen's kappas (k) were higher than 0.75 [46].

### Data Analysis

We ran three sets of linear mixed models via SPSS 19.0. The first analysis was performed via GLMM (Generalized Linear Mixed Model) to examine the effect of different variables on the presence/absence of yawn contagion. In this case a binomial distribution and a logit link function were used and the dependent variable was a binary term of whether yawn contagion occurred or not.

The second analysis was run via LMM (Linear Mixed Model) to examine the effect of different variables on the frequency of yawn contagion. The dependent scale variable was the relative frequency of yawn contagion by each responder (the observer) measured as the number of times such responder had yawned after a given trigger's yawn normalized on the number of occasions (number of times the observer had the opportunity to perceive a given trigger yawning).

For the third analysis we applied GLMM to examine the effect of different variables on the latency time in the yawn response. A multinomial distribution and a generalized logit link function were used and the dependent variable was a multinomial term expressing the time delay between trigger's and responder's yawn.

In all analyses, triggers and observers' identities (a personal code assigned to every subject) were entered as random factors (nominal variables).

We tested models for each combination involving the variables of interest, spanning from a single-variable model to a model including all the fixed factors (full model). To select the best model, we used the Akaike's Corrected Information Criterion (AICc), a measure for comparing mixed models based on the -2 (Restricted) log likelihood. The AICc corrects the Akaike's Information Criterion (AIC) for small sample sizes. As the sample size increases, the AICc converges to AIC. The model with a lower value of AIC was considered to be the best model.
